# Spectrophotometric Study of Charge-Transfer Complexes of Ruxolitinib with Chloranilic Acid and 2,3-Dichloro-5,6-dicyano-1,4-benzoquinone: An Application to the Development of a Green and High-Throughput Microwell Method for Quantification of Ruxolitinib in Its Pharmaceutical Formulations

**DOI:** 10.3390/molecules28237877

**Published:** 2023-11-30

**Authors:** Khalid A. Aljaber, Ibrahim A. Darwish, Abdullah M. Al-Hossaini

**Affiliations:** Department of Pharmaceutical Chemistry, College of Pharmacy, King Saud University, P.O. Box 2457, Riyadh 11451, Saudi Arabia

**Keywords:** ruxolitinib, charge-transfer complex, microwell spectrophotometry, quality control, green and high-throughput approach

## Abstract

Ruxolitinib (RUX) is a potent drug that has been approved by the Food and Drug Administration for the treatment of myelofibrosis, polycythemia vera, and graft-versus-host disease. This study describes the formation of colored charge-transfer complexes (CTCs) of RUX, an electron donor, with chloranilic acid (CLA) and 2,3-dichloro-5,6-dicyano-1,4-benzoquinone (DDQ), the π-electron acceptors. The CTCs were characterized using UV-visible spectrophotometry. The formation of CTCs in methanol was confirmed via formation of new absorption bands with maximum absorption at 530 and 470 nm for CTCs with CLA and DDQ, respectively. The molar absorptivity and other physicochemical and electronic properties of CTCs were determined. The molar ratio was found to be 1:1 for both CTCs with CLA and CTCs with DDQ. The site of interaction on RUX molecules was assigned and the mechanisms of the reactions were postulated. The reactions were employed as basis for the development of a novel green and one-step microwell spectrophotometric method (MW-SPM) for high-throughput quantitation of RUX. Reactions of RUX with CLA and DDQ were carried out in 96-well transparent plates, and the absorbances of the colored CTCs were measured by an absorbance microplate reader. The MW-SPM was validated according to the ICH guidelines. The limits of quantitation were 7.5 and 12.6 µg/mL for the methods involving reactions with CLA and DDQ, respectively. The method was applied with great reliability to the quantitation of RUX content in Jakavi^®^ tablets and Opzelura^®^ cream. The greenness of the MW-SPM was assessed by three different metric tools, and the results proved that the method fulfills the requirements of green analytical approaches. In addition, the one-step reactions and simultaneous handling of a large number of samples with micro-volumes using the proposed method enables the high-throughput analysis. In conclusion, this study describes the first MW-SPM, a valuable analytical tool for the quality control of pharmaceutical formulations of RUX.

## 1. Introduction

Ruxolitinib (RUX) is a small molecule that belongs to a class of drugs known as Janus kinase (JAK) inhibitors. It is primarily used for the treatment of certain blood disorders and inflammatory conditions (myelofibrosis, polycythemia vera, graft-versus-host disease, and vitiligo). RUX exerts its effects by targeting and inhibiting JAK enzymes, particularly JAK1 and JAK2. By inhibiting JAK1 and JAK2, RUX helps regulate the abnormal signaling associated with certain diseases. [1]. RUX is marketed as oral tablets under the brand names of Jakafi^®^ (Incyte Corporation, Wilmington, DE, USA) and Jakavi^®^ (Novartis, Basel, Switzerland). The Jakafi^®^ tablet was firstly approved for the treatment of myelofibrosis, a rare bone marrow disorder, on 16 November 2011 [2,3]. On 24 May 2019, the FDA approved Jakafi^®^ for steroid-refractory acute graft-versus-host disease in adult and pediatric patients 12 years and older [4]. On 22 September 2021, the FDA approved RUX for chronic graft-versus-host disease [5]. On 18 July 2022, the FDA approved RUX, as Opzelura^®^ cream, for the treatment of acute and nonsegmental vitiligo, a chronic autoimmune condition that causes patches of skin to lose pigment and turn milky white [6]. Opzelura^®^ is the first FDA-approved drug that can restore pigmentation in vitiligo patients. It is applied twice a day to affected areas, and satisfactory response may require treatment with Opzelura^®^ for more than 24 weeks. RUX functions by lowering an individual’s enhanced immune response [6]. 

While there are effective benefits of RUX in managing these conditions, there are some associated potential side effects. The most common side effects are thrombocytopenia, anemia, hepatic toxicity, infections, and non-melanoma skin cancer. The incidence, severity, and tolerability of these effects are dose-dependent, thus accurate dosing and dose adjustment are very important for achieving a potent and safe therapy with RUX [3,7]. To achieve this goal, a proper analytical method is required for the quality control of RUX-containing pharmaceutical formulations (tablets and cream). Few methods exist in the literature for quantitation of RUX in its formulations [8,9,10,11,12], and most are chromatography. These methods suffer from major drawbacks. These drawbacks include long analysis times because of the time-consuming/labor-intensive sample preparation steps, the equilibration of columns within the mobile phase before each analysis to ensure consistent and reproducible results, regular column maintenance, system suitability testing, challenges posed by the transferability of methods between different laboratories or instruments, and consumption of large volumes of costive and environmentally/health hazardous organic solvents in the mobile phase. These features limit the ability of method to meet the demands of high throughput in pharmaceutical analysis [13,14,15], and cause failure of the procedures to meet the green analytical approaches in pharmaceutical analysis [16,17,18,19]. Furthermore, all these methods were developed for Jakavi^®^ tablets, and no method was found in the literature for the quantitation of RUX in Opzelura^®^ cream. For these reasons, the development of an alternative green method with high throughput for quality control of both RUX’s pharmaceutical formulations (Jakavi^®^ tablets and Opzelura^®^ cream) was important.

Microwell spectrophotometry is a prominent eco-friendly/green approach in pharmaceutical analysis [20,21]. An essential benefit of this methodology is its utilization of smaller sample/reagent volumes compared to conventional practices in spectrophotometric methods. This not only minimizes waste generation but also reduces expenditure on reagents. Moreover, by employing microscale techniques, the amount of hazardous chemicals/solvents used in the laboratory can be decreased, contributing to enhanced sustainability [22,23]. The versatility of this technique allows for various applications in the pharmaceutical industry [24,25,26]. Microwell spectrophotometry lends itself well to automation using robotic systems, leading to improved efficiency, reduced errors, and time and labor savings in the laboratory. Automation also facilitates the adoption of renewable energy sources, such as solar and wind power, to decrease the carbon footprint of analytical chemical processes [23]. These advantages have significantly increased the popularity of this technique not only within the pharmaceutical industry but also in other domains [20,22].

This study describes the development of a microwell-spectrophotometric method (MW-SPM) for quantitation of RUX in Jakavi^®^ tablets and Opzelura^®^ cream. The method involved the microscale in-microwell formation of colored charge-transfer complexes (CTCs) through reactions of RUX, an electron donor, with chloranilic acid (CLA) and 2,3-dichloro-5,6-dicyano-1,4-benzoquinone (DDQ) reagents, the electron acceptors. Both reactions were conducted in transparent 96-well plates and the absorbances of each reaction’s solutions were measured by an absorbance microplate reader at 530 and 470 nm for reactions with CLA and DDQ, respectively. The proposed MW-SPM meets the requirements of high-throughput and green analytical approaches used in the pharmaceutical industry.

## 2. Results and Discussion

### 2.1. Strategy for Methodological Development

We were motivated to choose RUX as the target analyte for this study due to several factors. Firstly, RUX has been approved by the FDA and has shown clinical success and therapeutic benefits in the treatment of myelofibrosis, polycythemia vera, graft-versus-host disease, and nonsegmental vitiligo. Additionally, the lack of analytical methods for quality control of its pharmaceutical formulations has created an urgent need for accurate and simple methods to quantify RUX in its formulations. Considering the simplicity and wide availability of the spectrophotometric technique in quality control units of pharmaceutical companies, we decided to develop spectrophotometric methods for RUX. The technique offers, in addition to procedural simplicity, adaptability to automated analyzers, which can analyze a large number of samples for the quality control assessment of pharmaceutical formulations [27].

**Figure 1:**
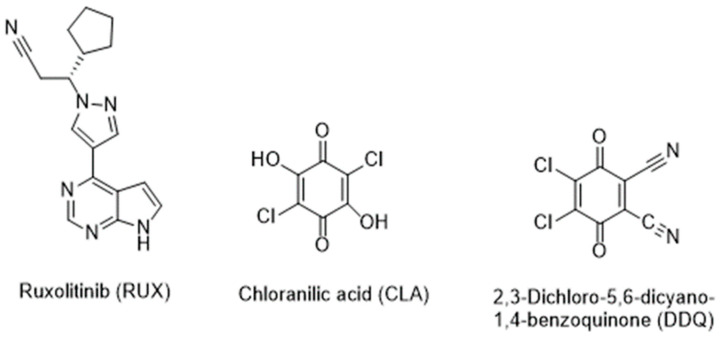
The chemical structures of ruxolitinib (RUX), chloranilic acid (CLA), and 2,3-dichloro-5,6-dicyano-1,4-benzoquinone (DDQ).

Considering the advantages of microwell-based analysis [20,21,22,23,24,25], the methodology was considered for this study. The formation of CTCs plays a crucial role in various scientific disciplines, including spectrophotometry-based pharmaceutical analysis [28,29,30,31]. The chemical structure of RUX (Figure 1) contains electron-donating nitrogen atoms, which have the potential capability to participate in the formation of CTCs with electron acceptors. Surprisingly, investigations of charge-transfer reactions of RUX have not been reported yet. Given this knowledge gap, the present study aimed to develop a CT-based spectrophotometric method for RUX. Among several polyhalo-/polycyanoquinone electron acceptors, CLA and DDQ (Figure 1) were identified as the most reactive acceptors as they demonstrated instantaneous reactions under very mild conditions [32,33]. Therefore, CLA and DDQ were selected in this study for the formation of CTCs with RUX and their employment in the development of a MW-SPM for the quantitation of RUX.

### 2.2. Reactions and Absorption Spectral Characteristics

**Figure 2:**
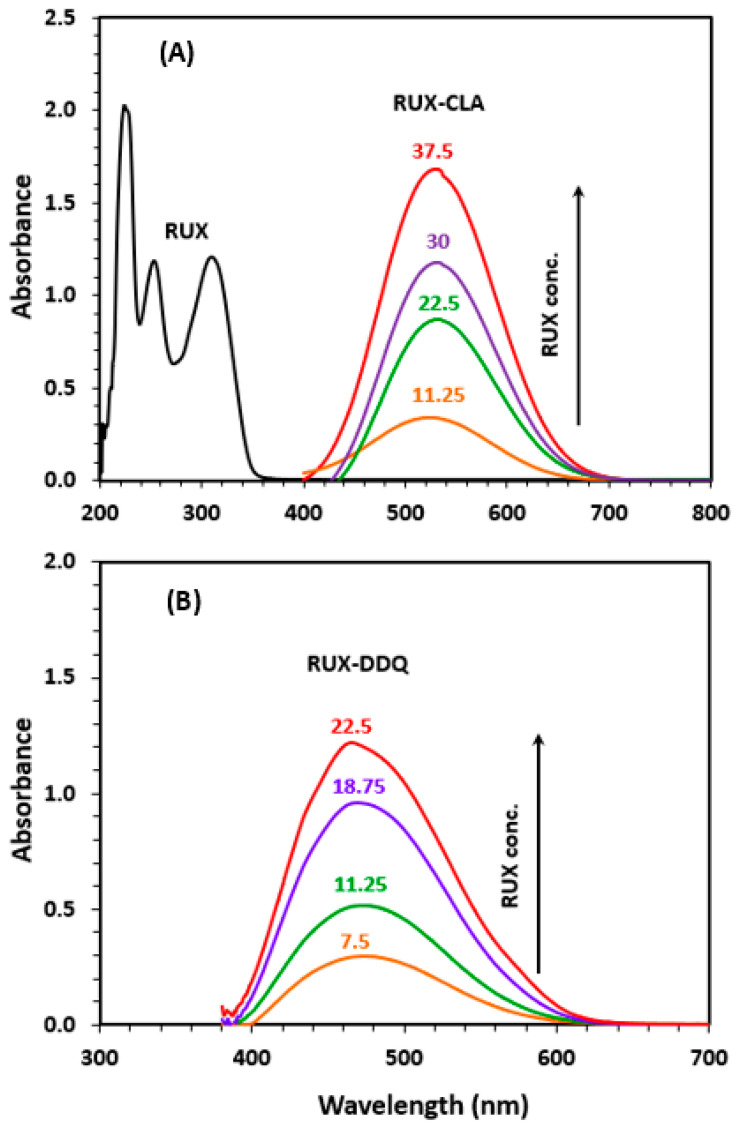
Panel (**A**) absorption spectra of RUX (30 µg/mL) and reaction mixtures of RUX and CLA (RUX-CLA) containing a fixed concentration of CLA (0.025%, *w*/*v*) and varying concentrations of RUX (11.25–37.5 µg/mL). Pane (**B**) absorption spectra of reaction mixtures of RUX and DDQ (RUX-DDQ) containing a fixed concentration of DDQ (0.025%, *w*/*v*) and varying concentrations of RUX (7.5–22.5 µg/mL). All solutions were in methanol. Figures on the spectral lines are the RUX concentrations used to generate the spectra.

The absorption spectrum of the RUX solution (30 µg/mL, in methanol) was recorded (Figure 2A), and it showed three distinct absorption peaks at 225, 253, and 310 nm. After mixing RUX solutions of varying concentrations (11.25–37.5 µg/mL) with a fixed concentration of CLA solution (0.025%, *w*/*v*), the reaction mixtures turned violet-colored. The absorption spectra of these reaction mixtures showed a new absorption band with an absorbance maximum at 530 nm (Figure 2A). The absorbances of this band increased as concentrations of RUX increased. The appearance of this new band and the dependence of its absorbance on RUX concentrations confirmed the occurance of the reaction between RUX and CLA. It was documented that CLA exists in two stable forms: a purple-colored form (HA^−^) and a pale violet form (A^2−^). In non-polar solvents (e.g, 1,4-dioxane), CLA showed absorption bands at 301 and 420 nm due to the π−π* transition within the electronic system of the quinonoid ring and the proton transfer between CLA and dioxane. It was also documented that the band at 420 nm was shifted bathochromically to 530 nm in the presence of basic molecules, which was attributed to the existence of the HA^—^ form of CLA. In polar solvents (e.g., acetonitrile and methanol), CLA imparts a purple color that is characterized by a weak absorption band at 530 nm. This band significantly increased following the reaction of CLA with electron donor molecules [34]. Since the reaction of RUX with CLA was conducted in the present study in methanol, it was concluded that the resulting violet reaction product of RUX with CLA corresponds to the HA^—^ form of CLA participating in the reaction with RUX. Similarily, after mixing varying concentrations of RUX solutions (7.5–22.5 µg/mL) with a fixed concentration of DDQ solution (0.025%, *w*/*v*), the reaction mixtures turned red-colored. The absorption spectra of these mixtures showed a new absorption band with an absorbance maximum at 470 nm (Figure 2B). In a similar vein, the absorbances of the band increased as the concentrations of RUX increased, revealing the occurance of reactions between RUX and DDQ.

The shape and pattern of the new absorption bands were identical to those of the radical anions of CLA and DDQ, as described in the literature [32,33]. The confirmation of the CT nature of the reactions was further supported by the disappearance of colors in both reaction mixtures upon their acidification with mineral acid (HCl). Building on these results, the reactions of RUX with both CLA and DDQ were presumed to be a CT reaction between RUX, the electron donor (D), and CLA and DDQ, the π-electron acceptors (A), proceeding in methanol (as an example of a polar solvent). Each reaction resulted in the formation of a CTC (D-A), which had dissociated and yielded the colored radical anion of the acceptor (A^•−^):

### 2.3. Characterization of CTCs

**Figure 3:**
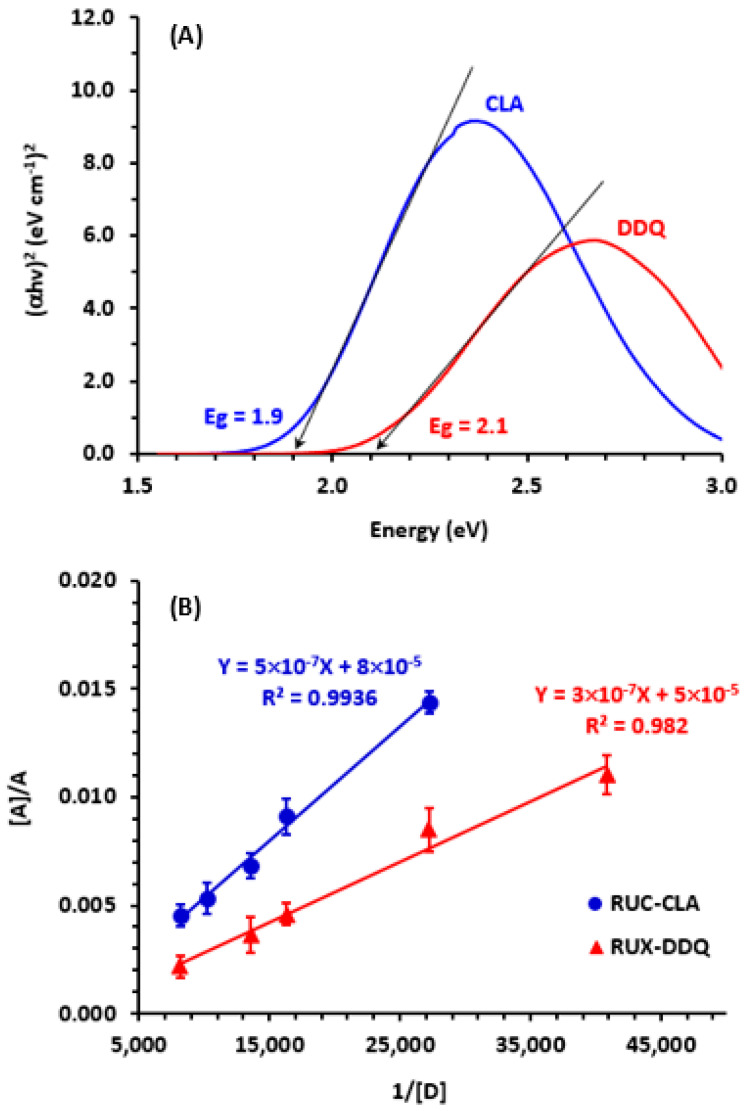
Panel (**A**) Tauc plots of energy (hυ) against (αhυ)^2^ for CTCs of RUX with CLA and DDQ in methanol solvent. Panel (**B**) Benesi–Hildebrand plots for formation of the CTCs of RUX with CLA and DDQ. Linear fitting equations and their determination coefficients (R^2^) are provided on the plot. [A], A, and [D] are the molar concentration of the acceptor reagent (CLA or DDQ), absorbance of the CTC, and molar concentration of RUX, respectively. The values are the means of 3 measurements ± SD.

The characteristics of CTCs of RUX with CLA and DDQ were determined in terms of their band gap energy (Eg) and other relevant electronic constants according to the procedures described in a previous study [35]. Eg of a CTC refers to the energy difference between the highest occupied molecular orbital (HOMO) and the lowest unoccupied molecular orbital (LUMO) of the complex. It represents the energy required to promote an electron from the HOMO to the LUMO. To determine the Eg of RUX CTCs with CLA and DDQ, a Tauc plot for each CTC was generated and used for deriving the Eg value (Figure 3A). The Eg values were found to be 1.9 and 2.1 eV for CTCs of CLA and DDQ, respectively. These low Eg values indicated that electron transfers from RUX to each of CLA and DDQ occurred easily without a need for high light energy, making the reactions suitable for applications to the development of spectrophotometric methods for RUX [36].

The other electronic constants/properties (Table 1) of CTCs of RUX with CLA and DDQ were determined. The high association constants and low standard free energy change of both CTCs with CLA and CTCs with DDQ, derived from Benesi–Hildebrand [37] plots (Figure 3B), indicate that the interactions between RUX and each of CLA and DDQ occurred easily, and the resulting CTCs were sufficiently stable. Furthermore, the high molar absorptivity (ε) obtained with both CLA and DDQ indicated their suitability for the development of sensitive spectrophotometric methods for RUX.

**Figure 4:**
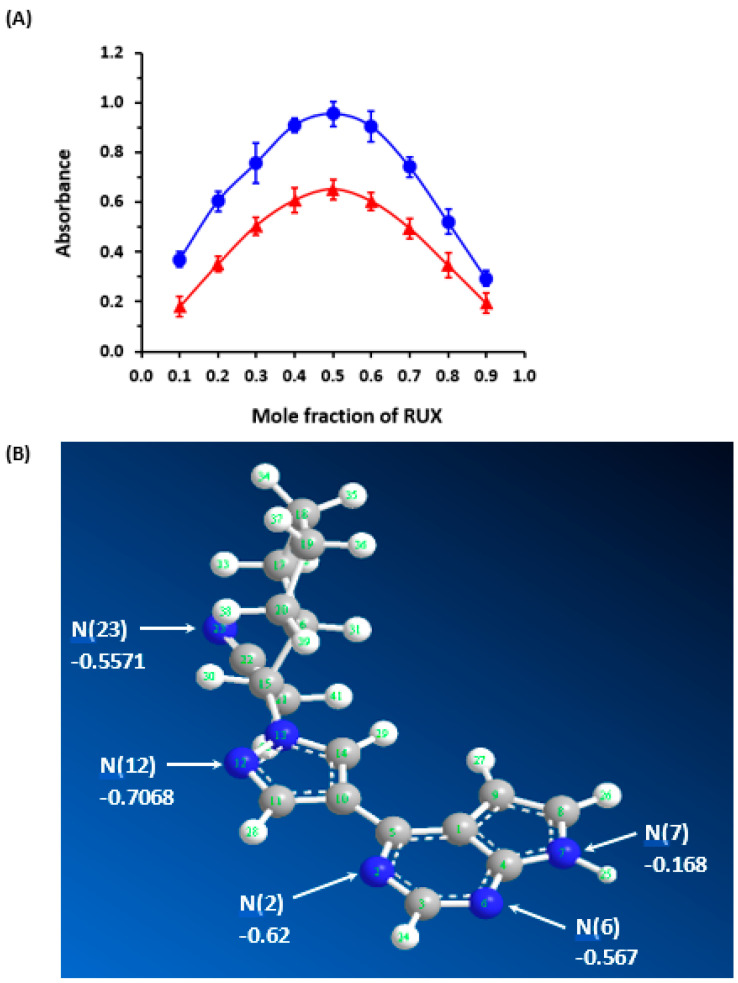
Panel (**A**) Job’s continuous variation plots for determination of the molar ratio of the CTCs of RUX with CLA (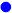) and DDQ (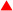). The values are the means of 3 measurements ± SD. Panel (**B**) a molecule of RUX with atom numbers, and the arrows pointing to the potential atoms for participation in the formation of CTCs, along with their partial charges. Negative sign denotes negative electron density.

**Figure 5:**
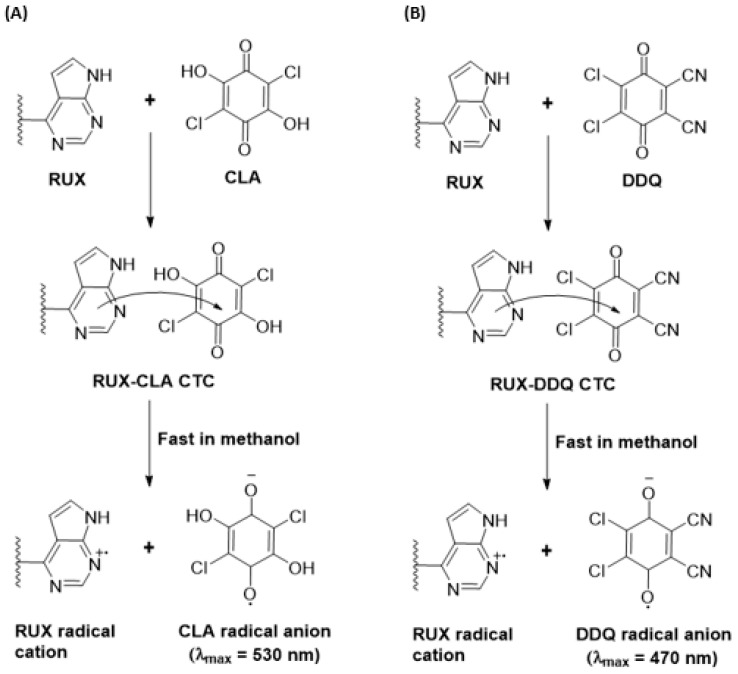
The schemes for formation of CTCs of RUX with CLA (**A**) and DDQ (**B**).

The molar ratios of CTCs of RUX with both CLA and DDQ were determined using Job’s method [38], and they were found to be 1:1 for both CTCs with CLA and CTCs with DDQ (Figure 4A). This ratio revealed that only one molecule of an acceptor contributed to the formation of the complex with one electron-donating RUX molecule. To distinguish a specific site among several accessible electron-donating sites on the RUX molecule (Figure 1), partial charges of each atom on the RUX molecule were calculated using the Molecular Orbital PACkage (MOPAC) software (Molecular Sciences Software Institute, Berkeley, CA, USA, version 16.0). The results are presented in Table 2, as elementary charges (e). The highest partial charges were found on nitrogen atoms number 2, 6, 12, and 23, indicating that one of them could participate in the formation of CTCs of RUX with CLA and DDQ. The summation of charges on nitrogen atoms numbers 6 and 7 (−0.567 + 0.168) was c0.735, which was higher than that of nitrogen atom number 12 of the pyrrole ring (−0.7068). These findings reveal that pyrimidin-4-yl-pyrazol moiety was the electron-donating site, via nitrogen atoms number 6 and 7, contributing to the formation of CTCs of RUX with both CLA and DDQ. According to these results, the reactions between RUX and each of CLA and DDQ were postulated to proceed as depicted in Figure 5.


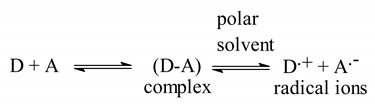


### 2.4. Development of the MW-SPM

In order to develop a MW-SPM and refine its procedure, factors affecting the reactions and absorbance of reaction mixtures were investigated. In these investigations, the reactions were conducted in 96-well transparent plates, and the absorbances were measured by an absorbance microplate reader at 530 and 470 nm for reactions with CLA and DDQ, respectively.

**Figure 6:**
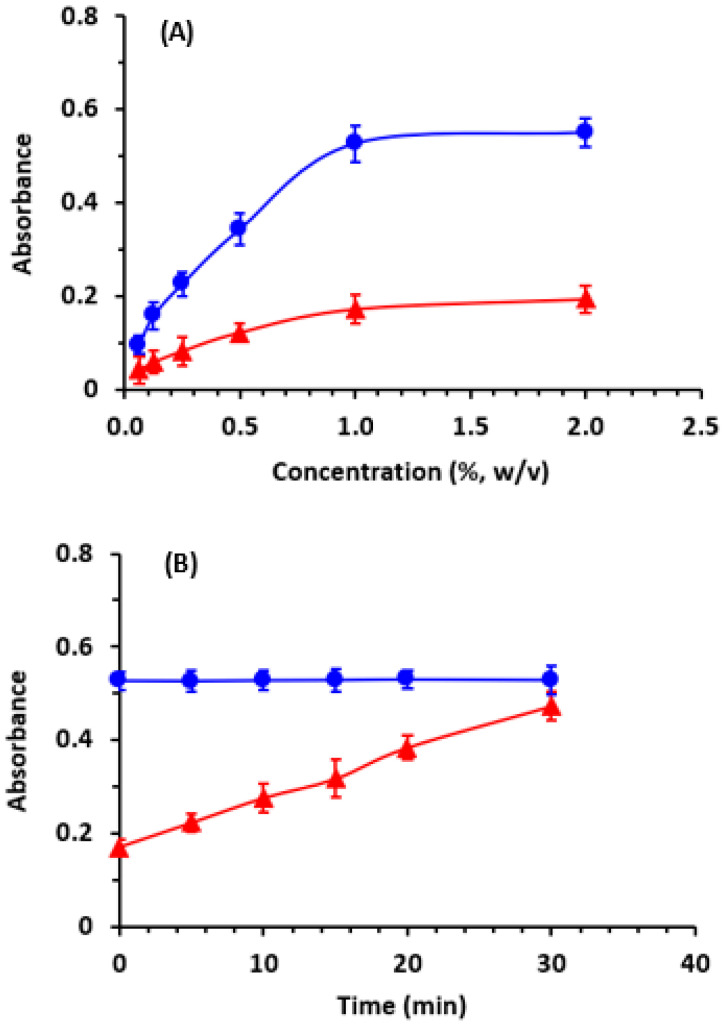
The effect of the acceptor reagent concentration (**A**) and time (**B**) on the CT reactions of RUX with CLA (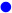) and DDQ (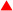). The points are the means of 3 determinations ± SD.

The effect of CLA and DDQ concentrations was investigated by conducting the reactions using different concentrations of both CLA and DDQ (ranging from 0.05 to 2% (*w*/*v*). The findings revealed that the absorbance of CTCs increased with increasing CLA and DDQ concentrations. The absorbances reached their maximum value when the concentrations of both CLA and DDQ was 1% (*w*/*v*), and beyond this concentration the absorbance did not change (Figure 6A). Consequently, a concentration of 1% (*w*/*v*) for both CLA and DDQ was selected for subsequent experiments.

At room temperature (25 ± 2 °C), the reaction of RUX with CLA was observed to take place immediately, and the resulting color intensity remained consistent for a duration of 30 min (Figure 6B). In the case of DDQ, the reaction proceeded slowly as indicated by a gradual continued increase in the absorbance with time (Figure 6B). The reaction of DDQ was not complete within 30 min; however, the absorbance obtained at the start of the reaction was adequate for development of a method with adequate sensitivity for the quantitation of RUX in its pharmaceutical formulations. In light of these conditions, and taking into account the fact that the main objective of the study was the development of a rapid method, measurements in the subsequent experiments were conducted within 5 min.

### 2.5. Validation of the MW-SPM

#### 2.5.1. Linear Range and Sensitivity

**Figure 7:**
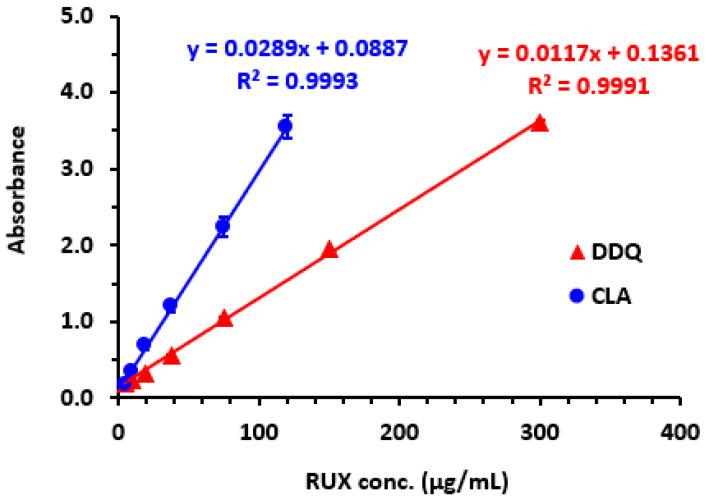
The calibration curves for quantitation of RUX by the MW-SPM via formation of CTCs with CLA and DDQ. The linear regression equations and their determination coefficients (R^2^) are given on calibration lines.

Calibration curves were created to establish relationships between RUX concentrations and their corresponding absorbances (Figure 7). The datasets were analyzed using linear regression with the least-squares method, resulting in derived fitting parameters (Table 3). The ranges of linearity spanned from 5 to 120 and from 5 to 300 μg/mL for the methods using CLA and DDQ, respectively, and their determination coefficients were 0.9993 and 0.9991, respectively. Table 3 provides the intercepts and slopes of the calibration lines. Following the ICH guidelines for validation of analytical procedures [39], the limit of detection (LOD) and limit of quantitation (LOQ) were determined using the formula: LOD = 3.3 SDa/b and LOQ = 10 SDa/b, where SDa and b were the standard deviation of the linear intercept and its slope, respectively. The LOD values were 2.5 and 4.2 μg/mL for CLA and DDQ methods, respectively, and the LOQ values were 7.5 and 12.6 μg/mL, respectively (Table 3).

#### 2.5.2. Precision and Accuracy

The proposed MW-SPM was utilized to analyze replicate samples of RUX solutions with low, medium, and high concentrations (Table 4). The readings obtained from these samples were used to calculate the relative standard deviations (RSD), which served as a measure of method precision. For both intra-day and inter-day precision, the RSD values did not exceed 2% for CLA and DDQ methods, respectively. These small RSD values indicate that the methods exhibited high precision. To assess the methods’ accuracy, recovery studies were conducted using the same RUX samples. As indicated in Table 4, the recovery values ranged from 99.4% to 101.6%, with error values in the range of −0.6–1.6%). These high recovery and low error values demonstrate high accuracy.

#### 2.5.3. Specificity and Interference

The proposed method’s specificity for the analysis of RUX tablets and cream without interference from the inactive excipients or other additives used for both formulations was evident for two primary reasons. Firstly, the measurements conducted in the assay were carried out in the visible region of the electromagnetic spectrum (530 and 470 nm for CLA and DDQ, respectively), which is far from UV-absorbing excipients/additives. The measurements at these wavelengths ensured that the excipients did not affect the accuracy of measurement for either tablets or cream. Secondly, the use of methanol for the dissolution and preparation of the tablet sample solution played a key role because methanol selectively dissolved RUX while leaving the excipients undissolved since they are insoluble in methanol. These two factors demonstrate the method’s specificity, allowing for accurate determination of RUX in the tablets and cream without interference from the inactive ingredients.

#### 2.5.4. Robustness and Ruggedness

The robustness of the MW-SPM was assessed for investigating the impact of minor variations in the method’s variables on its performance. These variables included the concentration of CLA and DDQ, and reaction times were modified by 20% from their optimal values, as outlined in Table 5. Notably, it was observed that slight deviations in these variables did not have significant effects on the results of either the CLA or DDQ method. The recovery values obtained were in the ranges of 98.2–101.8% and 98.6–101.8%, for CLA and DDQ methods, respectively. The precisions, expressed as RSD values, were in the ranges of 1.1–1.8% for both CLA and DDQ, respectively. These findings validate the suitability of the proposed method for routine analysis of RUX.

Additionally, the ruggedness of the method was evaluated by involving two different analysts to perform the procedures on three different days. The results obtained from the day-to-day variations exhibited reproducibility, as the RSD values did not exceed 2% for either the CLA or DDQ method. This further supports the reliability and consistency of the methods, even when conducted by different analysts on different occasions.

### 2.6. Analysis of Jakavi^®^ Tablets and Opzelura^®^ Cream

The successful applicability of the proposed method for analyzing RUX in its bulk form was evident from the acceptable validation results mentioned above. The method was employed to quantify RUX in both Jakavi^®^ tablets and Opzelura^®^ cream, using different concentrations as specified in Table 6. In Table 6, the determined label claim percentages are presented. The mean label claim percentages of Jakavi^®^ tablets were 100.8 ± 1.2 for reactions with both CLA and DDQ, and those of Opzelura^®^ cream were 99.7 ± 1.5 and 100.7 ± 1.1%, respectively.

### 2.7. Assessment of Greenness Levels of the MW-SPM

In general, microwell-based analytical methods assisted by microplate readers follow the principles of Green Analytical Chemistry (GAC) by virtue of their analytical processes being miniaturized. This approach enables the use of smaller sample and reagent volumes, resulting in reduced waste production compared to traditional techniques. To evaluate the environmental friendliness of the proposed method, three effective metric tools were utilized: AES (Analytical Eco-Scale) [40], GAPI (Green Analytical Process Index) [41], and AGREE (Analytical Greenness Evaluation) [42]. These tools offer a precise and comprehensive assessment of the sustainability of analytical procedures, and detailed information regarding their evaluation parameters and a presentation of results can be found in the corresponding published articles.

The results obtained from AES can be found in Table 7 for the CLA and DDQ methods using two reagents of the same chemical category and involving similar operating conditions. Regarding the amount of solvent and reagent involved, the use of 100 µL of solvent (methanol) and 100 µL of reagent (CLA and DDQ) resulted in a total of 2 penalty points (PPs), 1 for each. The hazardous impact of methanol and reagents contributed to a subtotal of 9 PPs.

The parameters related to energy consumption by the instrument (absorbance microplate reader) and occupational hazards did not get any PPs as they adhered to the guidelines set by GAC. Waste production and treatment parameters received a subtotal of 1 and 3 PPs, respectively. The score was assigned because the assay generated less than 1 mL of waste per sample and the waste was not treated. Consequently, the total PPs for the assay amounted to 13, yielding an Eco-Scale score of 88 (100−12). This high score (>75) indicates that the proposed MW-SPM exhibits an excellent level of eco-friendliness [40].

**Figure 8:**
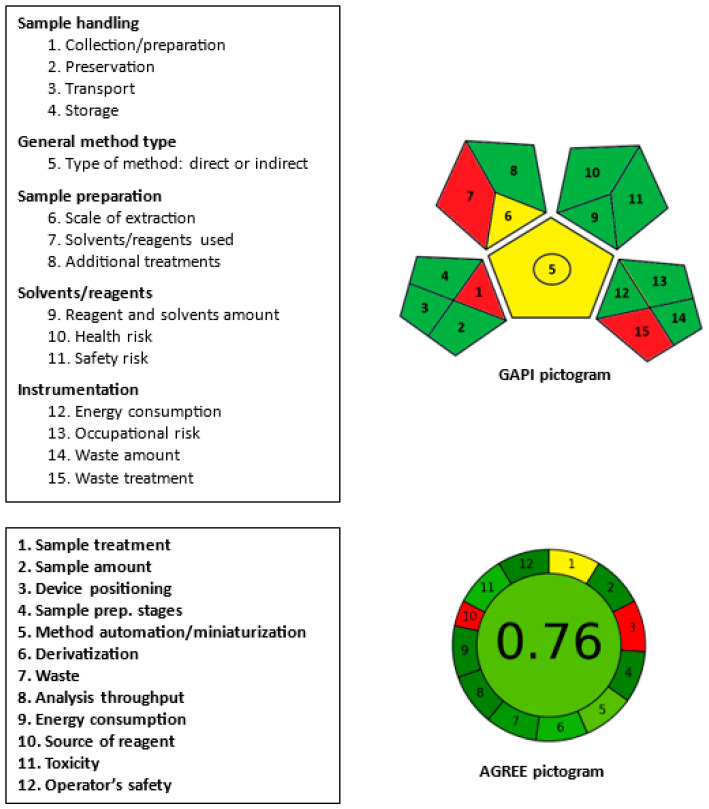
Results of the GAPI and AGREE metric tools for assessment of the greenness of the proposed MW-SPM for RUX.

The results obtained from the GAPI tool, which encompasses fifteen parameters (classified under five categories), are presented in a pictogram, which can be seen in Figure 8. Out of the fifteen parameters, three parameters (1, 7, and 15) are depicted in red in the pictogram. These parameters were assigned red for the following reasons: parameter 1 indicates that sample collection/preparation was conducted in an off-line manner, parameter 7 signifies the use of methanol for sample preparation, and parameter 15 reflects that the assay’s waste was not treated. Parameters 5 and 6 are assigned a yellow color since the assay is applicable to quantitative analysis and the extraction of samples was conducted on a microscale, respectively. The remaining parameters are represented in green as they meet the requirements of green procedures as per the guidelines of the tool [41].

The AGREE pictogram, visualizing the assessment results, is displayed in Figure 8. Parameter 1, which relates to sample treatment, is marked with a yellow color due to the manual execution of sample treatment. Parameter 3, associated with device positioning (on-line or off-line), and parameter 10, concerning the reagent source, are both depicted in red. The analysis was conducted off-line using a plate reader, and DDQ was utilized as a chemical source of reagent, respectively. The remaining parameters are assigned a green color. The total score obtained is 0.76 out of 1, indicating a high level of greenness for the assay.

In a conclusive summary, the results obtained from the three assessment tools provide conclusive evidence of the eco-friendliness of the proposed MW-SPM for RUX and its alignment with the principles of GAC.

### 2.8. Assessment of the MW-SPM

Microwell methods have gained significant recognition in pharmaceutical analysis due to their capacity to analyze and manipulate numerous individual entities simultaneously and with high throughput. These methods utilize arrays of microscale wells or compartments, where each well can accommodate a single entity, such as a target analyte molecule [13,14,15]. The throughput of the proposed MW-SPM was evaluated based on the use of 96-well plates and a specific reaction time of 5 min for both CLA and DDQ. An analyst was able to feasibly process a minimum of 5 plates as a batch simultaneously and comfortably. Under these conditions, a total of 2880 samples could be processed per hour (5 plates × 96 wells × 6 rounds/hour). The ability to process this number of samples (2880) indicates the high throughput of the proposed MW-SPM. This already high throughput could be further enhanced using different approaches. These approaches include employing plates with a greater number of wells, such as 384, 1536, or 3456 wells, and automating the process by employing automated systems or robots. These strategies can contribute to even higher throughput, enabling the analysis of a larger number of samples within a given timeframe.

## 3. Materials and Methods

### 3.1. Apparatus

The absorption spectra were recorded using a double-beam ultraviolet-visible spectrophotometer (V-530: JASCO Co. Ltd., Kyoto, Japan). For the measurement of absorbance, an absorbance microplate reader (ELx808: Bio-Tek Instruments Inc., Winooski, VT, USA) controlled with KC Junior software (version 20.0), provided with the instrument, was utilized.

### 3.2. Chemicals and Materials

RUX was acquired from Selleck Chemicals (Houston, TX, USA). The Jakavi^®^ tablets (Novartis, Basel, Switzerland; Batch No. SM822) labelled as containing 15 mg RUX per tablet and Opzelura^®^ cream (Incyte Corporation, Wilmington, DE, USA; Batch No. NDC-50881) labelled as 1.5% were generously provided by the Saudi FDA (Riyadh, Saudi Arabia). CLA and DDQ were purchased from Sigma-Aldrich Chemicals Co. (St. Louis, MO, USA). Fresh solutions of both CLA and DDQ (1%, *w*/*v*, in methanol) were prepared. Corning^®^ 96-well transparent polystyrene assay plates were obtained from Merck & Co. (Rahway, New Jersey, NJ, USA). Adjustable single- and 8-channel pipettes, as well as reagent reservoirs with cover lids for solution dispensing, were products of Merck KGaA (Darmstadt, Germany). Other materials were obtained from Fisher Scientific (Carlsbad, CA, USA).

### 3.3. Preparation of Standard RUX Solutions

RUX stock solution (2 mg/mL) was prepared by dissolving 20 mg of RUX in 10 mL methanol. This stock solution was diluted with methanol to acquire working solutions of RUX with concentrations suitable for the corresponding experiments. These concentrations were in the ranges of 5–120 and 5–300 μg/mL for CLA and DDQ, respectively.

### 3.4. Preparation of Solutions of Jakavi^®^ Tablets and Opzelura^®^ Cream

Ten tablets of Jakavi^®^ were pulverized into a fine powder. The precise quantity of this powder, which is equivalent to 20 mg of RUX, was transferred to a 25-mL calibrated flask. Approximately 20 mL of methanol was introduced, and the contents were thoroughly mixed to ensure complete dissolution of RUX from the tablet components. The flask’s contents were filtered, and the resulting filtrate was subsequently diluted with methanol to produce RUX solutions with concentrations ranging from 5 to 300 μg/mL. These solutions were then analyzed by the MW-SPM to determine their respective RUX concentrations.

For preparation of samples of Opzelura^®^ cream for analysis, 1 g of cream was transferred into a 25-mL flask. Approximately 20 mL of methanol was added, and RUX was extracted from the cream base by vigorously shaking the flask contents for 5 min. The flask was allowed to settle, allowing any insoluble particles to settle to the bottom. The clear supernatant liquid was filtered, and the filtrate solution was diluted with methanol to produce RUX solutions with concentrations ranging from 5 to 300 μg/mL. These solutions were then analyzed by the MW-SPM to determine their respective RUX concentrations.

### 3.5. Determination of Association Constant

Two series of RUX solutions containing varying concentrations were prepared in methanol. These concentrations were in the ranges of 3.67 × 10^−5^–1.22 × 10^−4^ M and 2.45 × 10^−5^–1.22 × 10^−4^ M for reactions with CLA and DDQ, respectively. Each solution of these varying concentrations reacted with a fixed concentration of CLA (4.79 × 10^−3^ M) and DDQ (4.41 × 10^−3^ M). The reaction mixtures were equilibrated for ~5 min at room temperature (25 ± 2 °C). The absorbances of the combined solutions were measured, and these values were utilized to construct the Benesi–Hildebrand plots. The plot establishes a relationship between 1/[D] and [A]/A, where [D], [A], and A represent the molar concentration of RUX, the molar concentration of the acceptor (CLA and DDQ), and the absorbance, respectively. Regression analysis was performed on the plots, and the obtained results were employed to computationally determine the association constant of the CTCs.

### 3.6. Determination of Reaction Molar Ratio

Job’s method was employed for determination of the molar ratio of RUX CTCs with both CLA and DDQ. Equimolar solutions (2 × 10^−3^ M) of RUX and each of CLA and DDQ were prepared. Varying volumes of RUX and the acceptor solutions were dispensed into microwells of the analysis plate used to make up different complementary volumes of RUX:acceptor (0:00, 25:175, 50:150, 100:100, 125:75, 150:50, 175:25, and 200:0). The absorbances of each well were measured and plotted as a function of the mole fraction of RUX. The generated Job’s plots were used for determining the molar ratio of the reactions between RUX and each of CLA and DDQ.

### 3.7. Procedures of the MW-SPM

100 µL aliquots of the RUX standard solution, Jakavi^®^ tablets, or Opzelura^®^ cream, which contained various concentrations of RUX ranging from 5 to 250 µg, were precisely dispensed into the wells of an analysis plate. Subsequently, 100 µL of a 1% (*w*/*v*) solution of either CLA or DDQ was added to each well, and the reaction was allowed to proceed for 5 min at room temperature (25 ± 2 °C). The absorbance of the solutions in each well was then measured using an absorbance microplate reader at 530 and 470 nm for analysis of reactions with CLA and DDQ, respectively.

## 4. Conclusions

The UV-visible investigation provided evidence of the formation of CTCs between RUX and each of CLA and DDQ. This was evident from the emergence of distinctive absorption bands at 530 and 470 nm for CTCs with CLA and DDQ, respectively. The molecular stoichiometry of both CTCs, with CLA and DDQ, was found to be 1:1. The association constants and other electronic constants/properties of the CTCs were determined, and the values revealed that the CTCs could be easily formed and are suitable for employment in the development of sensitive SPMs for quantitation of RUX. Computational charge calculations and molecular modelling were employed to suggest the site of interaction on RUX molecules that contributed to the formation of the CTCs with both CLA and DDQ. The formation of CTCs was employed as basis for the development of a MW-SPM for quality control of RUX-containing tablets and cream pharmaceutical formulations with high reliability. The proposed MW-SPM represents the first report ever describing a methodology for RUX. Noteworthy features of the method include its high-throughput capability, enabling the analysis of a large sample size within a short time frame. Additionally, the assay is environmentally friendly when utilized in pharmaceutical quality control laboratories, as it requires only a minimal amount of organic solvent for sample preparation. It is worth mentioning that the present study did not investigate the applicability of the proposed method for RUX in the presence of degradation impurities, which is considered a limitation of the proposed method. The proposed method has two main limitations. The first one is its sensitivity, which is not high enough to determine RUX in biological specimens (plasma and urine). The second one is its use of organic media for the reaction, which limits the application of the method to the analysis of RUX in aqueous samples.

## Figures and Tables

**Table 1:** Electronic constants/properties of the CTCs of RUX with CLA and DDQ.

Constant/Property	Value	
	CLA	DDQ
Association constant, *K* (L/mol)	1.47 × 10^2^	1.37 × 10^2^
Molar absorptivity, ε (L/mol/cm)	1.29 × 10^4^	2.07 × 10^4^
Ionization potential, I_p_ (eV)	8.65	9.02
Energy, hν (eV)	1.9	2.1
Resonance energy, R_N_ (eV)	0.32	0.52
Transition dipole moment, µ _(Debye)_	2.35	3.76
Oscillator strength, *f*	9.05	14.48
Standard free energy change, ΔG^0^ (J/mol)	−12.36	−12.77

**Table 2:** Atom types, numbers, and their calculated partial charges on the RUX molecule.

Atom Number	Atom Type	Partial Charge (e)
C(1)	Aromatic 5-ring carbon	0
N(2)	Aromatic nitrogen with s lone pair	−0.62
C(3)	Aromatic 5-ring carbon	0.47
C(4)	Aromatic carbon	0.341
C(5)	Aromatic carbon	0.364
N(6)	Aromatic nitrogen with s lone pair	−0.567
N(7)	Enamine or aniline nitrogen, delocalized lone pair	−0.168
C(8)	Aromatic 5-ring carbon	−0.066
C(9)	Aromatic 5-ring carbon	−0.15
C(10)	Aromatic 5-ring carbon	−0.054
C(11)	Aromatic 5-ring carbon	0.1388
N(12)	Aromatic 5-ring nitrogen	−0.7068
N(13(	Aromatic 5-ring nitrogen with p lone pair	0.314
C(14)	Aromatic 5-ring carbon	−0.3016
C(15)	ALKYL CARBON, SP3	0.2556
C(16)–C(20)	Alkyl carbon, SP3	0
C(21)	Alkyl carbon, SP3	0.2
C(22)	Cyano carbon	0.3571
N(23)	Triply bonded nitrogen	−0.5571
H(24)	Hydrogen attached to carbon	0.15
H(25)	Hydrogen on nitrogen in pyrrole	0
H(26)–H(29)	Hydrogen attached to carbon	0.15
H(30)–H(41)	Hydrogen attached to carbon	0

**Table 3:** The regression and statistical parameters for quantitation of RUX by the proposed MW-SPM via formation of CTCs with CLA and DDQ ^a^.

Parameter	Value	
	CLA	DDQ
Linear range (µg/mL)	5–120	5–300
Intercept	0.0887	0.1361
Slope	0.0289	0.0117
Determination coefficient (R^2^)	0.9993	0.9991
LOD (µg/mL)	2.5	4.2
LOQ (µg/mL)	7.5	12.6

^a^ The number of determinations was 3.

**Table 4:** The precision and accuracy of the MW-SPM for quantitation of RUX via formation of CTCs with CLA and DDQ.

RUX Concentration (µg/mL)	Intra-Day (*n* = 3)		Inter-Day (*n* = 6)	
	Recovery (% ± RSD)	Error (%)	Recovery (% ± RSD)	Error (%)
CLA				
20	100.5 ± 1.4	0.5	101.6 ± 1.4	1.6
60	99.6 ± 1.6	−0.4	99.4 ± 1.8	−0.6
100	101.4 ± 1.2	1.4	100.8 ± 1.1	0.8
DDQ				
40	100.5 ± 1.3	0.5	100.4 ± 1.4	0.4
150	99.4 ± 1.1	−0.6	99.8 ± 1.6	−0.2
250	101.2 ± 0.9	1.2	101.2 ± 1.2	1.2

**Table 5:** The robustness and ruggedness of the proposed MW-SPM for determination of RUX via formation of CTCs with CLA and DDQ.

Parameters	Recovery (% ± RSD) ^a^	
	CLA	DDQ
Robustness		
CLA concentration (%, *w*/*v*)		
0.8	99.6 ± 1.3	100.4 ± 1.2
1.2	100.4 ± 1.2	99.2 ± 1.6
DDQ concentration (%, *w*/*v*)		
0.8	98.2 ± 1.4	101.6 ± 1.5
1.2	101.3 ± 1.8	99.4 ± 1.1
Reaction time (min)		
3	101.8 ± 1.6	99.6 ± 1.4
7	100.5 ± 1.8	98.5 ± 1.5
Ruggedness		
Analyst-to-analyst		
Analyst-1	100.2 ± 1.1	99.5 ± 1.5
Analyst-2	99.4 ± 1.2	101.6 ± 1.7
Day-to-day		
Day-1	101.2 ± 1.3	98.4 ± 1.4
Day-2	98.6 ± 1.7	101.8 ± 1.5
Day-3	100.6 ± 1.8	99.2 ± 1.3

^a^ The values are the means of three determinations.

**Table 6:** The applications of the MW-SPM for quantitation of RUX in Jakavi^®^ tablets and Opzelura^®^ cream via formation of CTCs with CLA and DDQ.

Nominated RUX Concentration (µg/mL)		Label Claim (% ± RSD) ^a^	
		CLA	DDQ
Jakavi^®^ tablets			
25		99.4 ± 1.2	101.2 ± 1.2
50		101.8 ± 1.4	99.6 ± 1.5
100		101.1 ± 1.7	100.4 ± 1.8
250			101.2 ± 1.2
	Mean	100.8 ± 1.2	100.6 ± 0.8
Opzelura^®^ cream			
25		99.5 ± 1.4	101.5 ± 0.8
50		101.3 ± 1.2	99.2 ± 1.2
100		98.4 ± 1.8	100.7 ± 1.5
250			101.5 ± 0.9
	Mean	99.7 ± 1.5	100.7 ± 1.1

^a^ The values are the means of 3 determinations ± SD.

**Table 7:** Analytical Eco-Scale for assessing the greenness of the proposed MW-SPM for the quantitation of RUX via formation of CTCs with CLA and DDQ.

Eco-Scale Score Parameters	Penalty Points (PPs)
Amount of solvent/reagent	
Solvent: <1 mL (mL (g) per sample)	1
Reagent: <1 mL (mL (g) per sample)	1
	∑ = 2
Hazard of solvent/reagent	
Solvent: methanol	3
Reagent: CLA and DDQ	3
	∑ = 6
Instrument: Energy used (kWh per sample)	
Microplate reader	0
	∑ = 0
Occupational hazardous	
Analytical process hermetic	0
Emission of vapors and gases into the air	0
	∑ = 0
Waste	
Production (<1 mL (g) per sample)	1
Treatment (No treatment involved)	3
	∑ = 4
Total PPs	12
Eco-Scale score	88

## Data Availability

Data are contained within the article.
